# Epigenetic and non-epigenetic mode of SIRT1 action during oocyte meiosis progression

**DOI:** 10.1186/s40104-019-0372-3

**Published:** 2019-08-09

**Authors:** Jan Nevoral, Lukas Landsmann, Miriam Stiavnicka, Petr Hosek, Jiri Moravec, Sarka Prokesova, Hedvika Rimnacova, Eliska Koutna, Pavel Klein, Kristyna Hoskova, Tereza Zalmanova, Tereza Fenclova, Jaroslav Petr, Milena Kralickova

**Affiliations:** 1 Biomedical Center, Faculty of Medicine in Pilsen, Charles University, alej Svobody 1655/76, 323 00 Pilsen, Czech Republic; 2 Department of Histology and Embryology, Faculty of Medicine in Pilsen, Charles University, Karlovarska 48, 301 66 Pilsen, Czech Republic; 3 Faculty of Science, Charles University, Albertov 2038/6, 128 00 Prague, Czech Republic; 4 Faculty of Agriculture, Food and Natural Resources, Czech University of Life Sciences in Prague, Kamycka 129, 165 00 Praha-Suchdol, Czech Republic; 5 Institute of Animal Science, Pratelstvi 815/107, 104 00, Prague 10-Uhrineves, Czech Republic

**Keywords:** Epigenetics, Histone code, *In vitro* maturation, Oocyte, SIRT1, Sirtuin 1

## Abstract

**Background:**

SIRT1 histone deacetylase acts on many epigenetic and non-epigenetic targets. It is thought that SIRT1 is involved in oocyte maturation; therefore, the importance of the ooplasmic SIRT1 pool for the further fate of mature oocytes has been strongly suggested. We hypothesised that SIRT1 plays the role of a signalling molecule in mature oocytes through selected epigenetic and non-epigenetic regulation.

**Results:**

We observed SIRT1 re-localisation in mature oocytes and its association with spindle microtubules. In mature oocytes, SIRT1 distribution shows a spindle-like pattern, and spindle-specific SIRT1 action decreases α-tubulin acetylation. Based on the observation of the histone code in immature and mature oocytes, we suggest that SIRT1 is mostly predestined for an epigenetic mode of action in the germinal vesicles (GVs) of immature oocytes. Accordingly, BML-278-driven trimethylation of lysine K9 in histone H3 in mature oocytes is considered to be a result of GV epigenetic transformation.

**Conclusions:**

Taken together, our observations point out the dual spatiotemporal SIRT1 action in oocytes, which can be readily switched from the epigenetic to non-epigenetic mode of action depending on the progress of meiosis.

**Electronic supplementary material:**

The online version of this article (10.1186/s40104-019-0372-3) contains supplementary material, which is available to authorized users.

## Background

SIRT1, a mammalian homologue of yeast Sir2, belongs to NAD^+^-dependent histone deacetylases (also called sirtuins, SIRT1–7) [1]. SIRT1 shows the ability to deacetylate both epigenetic and non-epigenetic targets; therefore, SIRT1 molecular action leads to regulation of the cell cycle, apoptosis, and oxidative stress response, thereby influencing cell viability and senescence [2–4]. Resveratrol and several other polyphenolic compounds have been identified as sirtuin-activating, and their positive effect on oocyte viability was due to sirtuin activation [5–7]. However, an exact mechanism of SIRT1 action in oocytes has not been studied and, therefore, practical use of SIRT1-stimulating compounds remains to be limited.

Mammalian oocytes represent a unique model for the study of cell cycle regulation. Oocyte meiosis is synchronised in the G2/prophase and arrested for several years or decades in domestic animals and humans, respectively. Meiosis re-initiation, accompanied by nuclear envelope breakdown (NEBD) of germinal vesicles, is followed by further meiotic progression and finally, a mature oocyte is arrested again in metaphase of the second meiotic round (metaphase II), where the oocyte is predestined for fertilisation. The oocyte meiotic maturation is ingeniously orchestrated by the machinery of enzymes responsible for post-translational modifications (PTMs) of protein targets (summarised by Madgwick and Jones [8]). In addition to the well-known protein phosphorylation balanced by phosphatases/kinases [9–12], sirtuins represent an impactful protein-modulating acetylation of many cytoskeletal and/or regulatory proteins [13–16]. Moreover, SIRT1-driven deacetylation is involved in epigenome establishment, and SIRT1 represents an epigenetic factor affecting male germ cells [17] as well as early embryos [18]. Accordingly, SIRT1 involvement in oocyte epigenome modulation is taken into consideration.

There are many direct epigenetic SIRT1 substrates, i.e., histone lysine (K) residues, such as H3K9, H4K16 and others [19–21]. In a previous study, we revealed a SIRT1-modified histone code favouring histone H3K9 methylation in one-cell zygote [18]. This histone modification represents a relevant marker of SIRT1 activity through the associated signalling of SUV39H1 methyltransferase and MDM2 E3-ubiquitin ligase [18, 22]. Therefore, oocyte SIRT1 is considered to be essential for gametogenesis, arguably including oocyte meiotic maturation, for fertilisation and subsequent embryogenesis [17, 23, 24]. Additionally, the study of SIRT1 in oocyte meiosis provides relevant knowledge of the cell cycle of general medical significance.

In this study, our observations point out the involvement of SIRT1 in oocyte meiosis via epigenetic and non-epigenetic factors, based on affected targets. These results are the first to describe the benefits of a specific SIRT1 activator, BML-278, for the chromatin integrity of non-interphase cells through the revealed molecular mechanism. Moreover, our experiments show that pharmacological SIRT1 activation is a possible way to improve the viability of oocytes.

## Methods

### Animals

All animal procedures were conducted in accordance with Act No. 246/1992 Coll., on the Protection of Animals against Cruelty, under supervision of the Animal Welfare Advisory Committee at the Charles University, Faculty of Medicine in Pilsen, and approved by the Animal Welfare Advisory Committee at the Ministry of Education, Youth and Sports of the Czech Republic.

Six- to eight-week-old ICR female mice were maintained in a facility with a 12 h light: 12 h dark photoperiod, a temperature of 21 ± 1 °C and a relative humidity of 60% and had free access to food and water throughout the period of the study. Females were administered with i.p. 5 IU PMSG, and the experiment was terminated 48 h later for isolation of immature GV (germinal vesicle) oocytes. To obtain *in vivo* mature oocytes, PMSG-treated females were administered with 5 IU hCG, and cumulus-oocyte complexes were flushed from oviducts 16 h later.

### Chemicals

All chemicals were purchased from Sigma-Aldrich (St. Louis, MO, USA), if not otherwise stated. BML-278 (Abcam, Cambridge, UK; Cat. No. ab144536), a selective SIRT1 activator (EC_50_ = 1 μmol/L vs. EC_50_ 25 and 50 μmol/L for SIRT2 and SIRT3, respectively), and sirtinol, selective SIRT1 and SIRT2 deacetylase inhibitor (Abcam, ab141263), were used in this study. Moreover, BML-278 activity was compared with resveratrol (Abcam; ab120726), non-selective sirtuin activator, using a fluorometric SIRT1 Activity Assay Kit (Abcam; ab156065), in accordance with manufacturer’s instructions.

### *In vitro* maturation

Ovaries were dissected and immature fully grown oocytes at GV stage were isolated and manipulated in M2 medium supplemented with 100 μmol/L isobutyl-methylxanthine (IBMX). Fully grown and cumulus cell-free GV oocytes with intact ooplasm were placed into M16 medium containing 100 μmol/L IBMX for 1 h, followed by *in vitro* maturation in IBMX-free M16 for 16 h at 37 °C and 5% CO_2_. For the elucidation of the SIRT1 activation effect on the quality of mature oocytes, the culture medium was supplemented with BML-278 to final concentrations of 0.125, 0.25 and 0.5 μmol/L during oocyte meiosis progression. Alternatively, GV oocytes were treated with BML-278 in M16-IBMX for 16 h, and the effect of SIRT1 activation on GV chromatin was studied. In all treatment studies, BML-278 was dissolved in DMSO, and its concentration in M16 did not exceed 0.1% (*v*/*v*), therefore, a vehicle control (VC) consisting of 0.1% DMSO was included. Concurrently, untreated *in vitro*-matured oocytes were incubated with 10 μmol/L Taxol (in 0.1% DMSO, *v*/*v*), an anti-microtubule depolymerising agent, for 45 min at 37 °C. All oocytes were processed for immunocytochemistry as described below.

### Fixation and immunocytochemistry

Oocytes at all stages were fixed in two ways: either i) in 4% paraformaldehyde in PBS with 0.1% polyvinyl-alcohol (PVA), 30 min for at room temperature, or alternatively, ii) for H3K9me3, H3K4me2 imaging, in PFA-TX-100 for 15 min, at 37 °C, following permeabilisation in 0.03% Tween 20 in PBS-PVA for 60 s at 37 °C. Subsequently, all oocytes were equally permeabilised in PBS containing 0.04% Triton X-100 and 0.3% Tween 20, for 15 min. Thereafter, oocytes were blocked in 1% BSA in PBS with Tween 20 for 15 min. The 1 h incubation of oocytes with specific antibodies (all diluted 1:200, if not otherwise noted) followed: anti-SIRT1 (Abcam; ab104833; 1:200), anti-SIRT2 (Abcam; ab51023, 1:100), anti-α tubulin (Cell Signaling Technology, Leiden, Netherlands; #2144; 1:200), anti-acetylated α-tubulin (Abcam; ab24610; 1:200), anti-H3K9me2/3 (Abcam; ab184677; 1:200), anti-H3K4me2 (Abcam; ab7766; 1:200), and anti-ubiquitinated (K119) H2A (H2AK119ub; Cell Signaling Technology; D27C4; 1:200). Thereafter, washing and 1 h incubation with the cocktail of anti-mouse-AlexaFluor 488 and anti-rabbit-AlexaFluor 647 (1:200), respectively, were used. Concurrently with washing after the cocktail of secondary antibodies, phalloidin (Thermo Fisher Scientific, Waltham, MA, USA; 1:200) was applied for 15 min for β-actin visualisation. Stained oocytes were mounted onto slides in a Vectashield medium with 4′6´-diamidino-2-phenylindole (DAPI; Vector Laboratories Inc., Burlingame, CA, USA). Images were acquired using spinning disk confocal microscope Olympus IX83 (Olympus, Germany) and VisiView® software (Visitron Systems GmbH, Germany).

### TUNEL assay

Fixed oocytes were permeabilised in 0.1% Triton X-100 in PBS containing 0.05% NaN_3_ for 40 min. Oocytes were treated with fluorescein-conjugated dUTP and the terminal deoxynucleotidyl transferase enzyme (In Situ Cell Death Detection Kit, Cat. No. 11684795910, Roche, Mannheim, Germany), for 1 h in the dark at 37 °C, in accordance with the assay protocol. Positive control (PC) was prepared using DNase I kit (AMP-D1, Sigma-Aldrich). Finally, oocytes were mounted as mentioned above, and chromatin was visualised. Images were acquired as described above.

### Image analysis and colocalisation

Negative controls were performed by omitting specific antibodies and these slides were processed at comparable settings. Immuno- and TUNEL-stained oocytes were subjected to measurement of, integrated density’ (expressing signal intensity) of appropriate colour channels using ImageJ software (NIH, Bethesda, CA, USA). Nuclear signal intensities were scaled by signal intensity of corresponding ooplasms. Thereafter, the values of integrated density were related to control oocytes (VC = 1). JACoP (Just Another Co-localisation Plugin) approach for colocalisation of SIRT1 with spindle α-tubulin was used (according to Bolte and Cordelieres [25]). The Costes’ randomisation (Costes’ rand), modifying Pearson’s coefficient *R*_*r*_ according to Costes et al. [26], and Manders’ overlap coefficients (*R*, *M1* and *M2*) were used for estimation of colocalisation and overlap. Colocalisation analysis was performed on oocyte spindles used as the region of interest (ROI).

### Western blotting

Oocytes were collected and lysed in Laemmli buffer containing Triton-X-100 (0.003%, *v*/*v*) and SDS (0.001%, *v*/*v*), enriched with Complete Mini Protease Inhibitor Cocktail (Roche, Switzerland). Samples were boiled and subjected to SDS-PAGE electrophoresis in precast gradient gels and blotted using Trans-Blot TurboTM Transfer System onto a PVDF membrane (Bio-Rad Laboratories, Steenvoorde, France). After blocking in 5% non-fat milk in TBS with 0.5% Tween-20 (TBS-T) overnight at 4 °C, the membrane was incubated with mouse monoclonal anti-SIRT1 (1:1,000). Mouse monoclonal anti-β-actin loading-control antibody (Santa-Cruz Biotechnology, Inc., UK; sc-47778; 1:1,000) was used under the same conditions. Subsequently, the membrane was incubated with horseradish peroxidase (HRP)-conjugated goat anti-mouse or anti-rabbit IgG in TBS-T (Thermo Fisher Scientific; 1:10,000) for 1 h at room temperature. Proteins with adequate molecular weight were detected using the ECL Select Western Blotting Detection Reagent (GE Healthcare Life Sciences, Amersham, UK) and visualised by ChemiDocTM MP System (Bio-Rad Laboratories, Steenvoorde, France).

### Statistical analysis

Data from three independent experiments were processed and analyzed. Because of their significant non-normality (Shapiro-Wilk test) the data are represented by medians with appropriate quantiles and a non-parametric method, i.e. Kruskal-Wallis ANOVA, was used for the comparison of the study groups. In case of a significant overall finding, the differences between individual group pairs were assessed by a post hoc test, using multiple comparisons of mean ranks. Because of an asymmetry of the data distribution, logarithmic scale was used in the boxplots. The data were processed with Statistica Cz 12 (StatSoft, USA). The level of statistical significance was set at α = 0.05 and two-tailed *P* values are indicated.

## Results

### SIRT1 re-localisation during the progress of oocyte meiosis

**Fig. 1:**
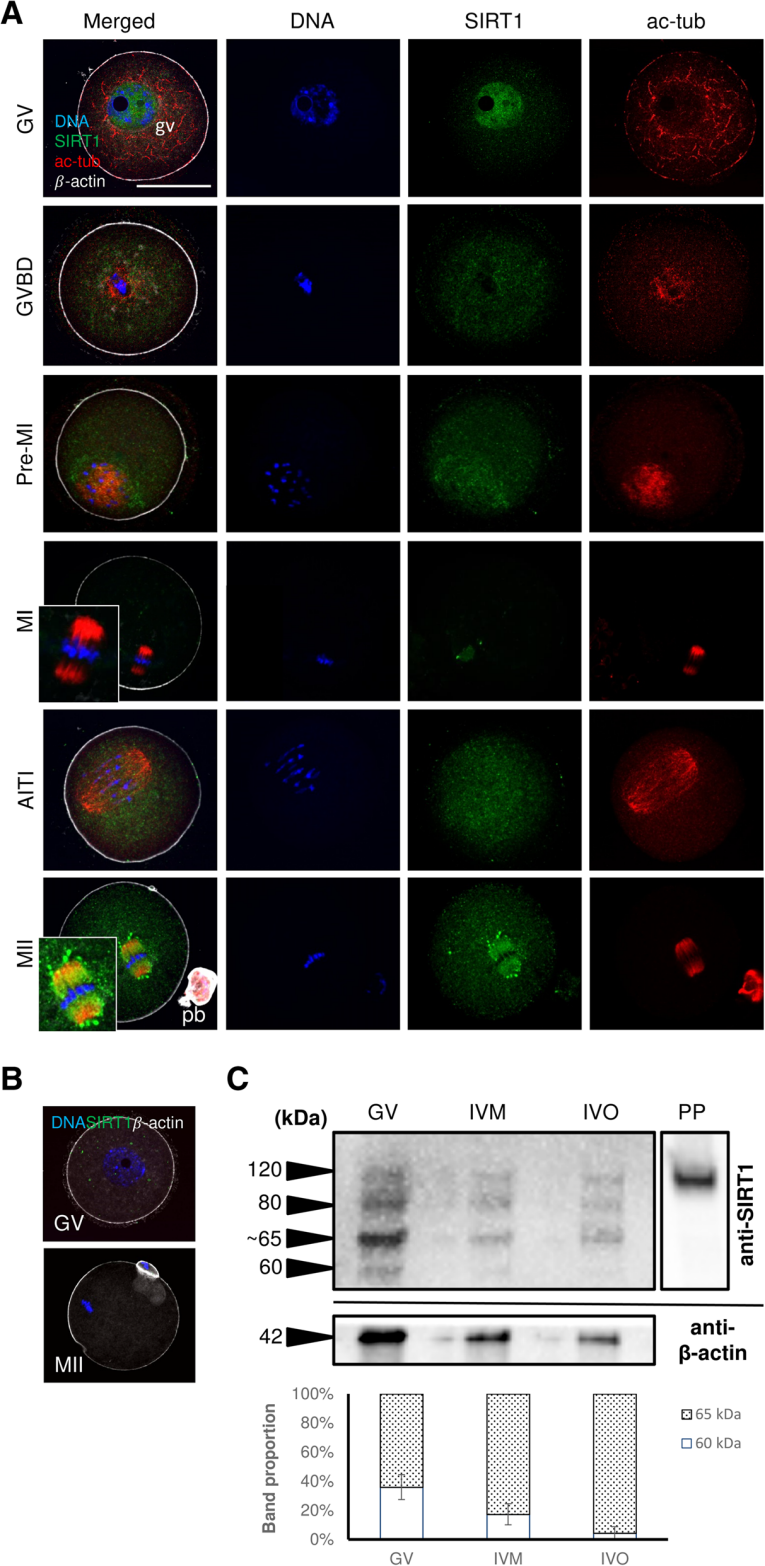
SIRT1 subcellular re-localisation during oocyte meiosis. **a** SIRT1 in immature GV oocytes, with visible germinal vesicle (gv), and *in vitro* produced oocytes at different stages, i.e., NEBD (nuclear envelope breakdown), pre-MI (pre-metaphase I), MI, AITI (anaphase-telophase transition) and MII (metaphase II) oocytes with extruded polar body (pb). Scale bar represents 50 μm. **b** Negative control of immunostaining where the primary antibody was omitted. **c** Immunoblotting of SIRT1 (60–120 kDa) and β-actin (42 kDa), in different oocytes (GV, IVM, IVO), including the proportion of 60 and 65 kDa bands (min – max values are indicated). Approximately 200 lysed oocytes were loaded per lane. PP: pure SIRT1 protein. The full-length blot is presented in Additional file 1: Figure S1.3)

In this experiment, we immunolabelled SIRT1 and described its subcellular localisation in mouse oocytes matured *in vitro*. For better visualisation of mature oocytes and their meiotic progress, α-tubulin and β-actin were co-immunolabelled. SIRT1 was exclusively located in germinal vesicles (GVs) of immature oocytes, and only a weak signal in ooplasm was obvious. As soon as the meiosis was re-initiated, SIRT1 was dramatically re-localised into the ooplasm of NEBD oocytes. In contrast to the spindles of metaphase I oocytes, where the SIRT1 signal almost disappeared, SIRT1 showed a spindle-like pattern in metaphase II oocytes (Fig. 1a). The binding specificity of anti-SIRT1 antibody (ab104833) against SIRT1 protein (Q923E4, UniProtKB) was verified by primary antibody omitting (Fig. 1b) and confirmed by Western blotting (Fig. 1c). SIRT1 expression was verified in GV oocytes, and *in vitro* (IVM) and *in vivo* (IVO) matured oocytes. An expected 120-kDa SIRT1 band as well as a ~ 80 kDa one (presumed 75 kDa fragment [27]) were detected, in accordance with the antibody manufacturer and UniProtKB database. Additionally, a 60-kDa and ~ 65 kDa bands were observed, for which a SIRT1 isoform 2 (59.9 kDa) was considered. Interestingly, the 60 kDa bands disappeared in matured oocytes while the 65 kDa bands remained (Fig. 1c). β-actin (42 kDa) was used as an internal standard.

### SIRT1 distributes in a spindle-like pattern when the oocyte matured

**Fig. 2:**
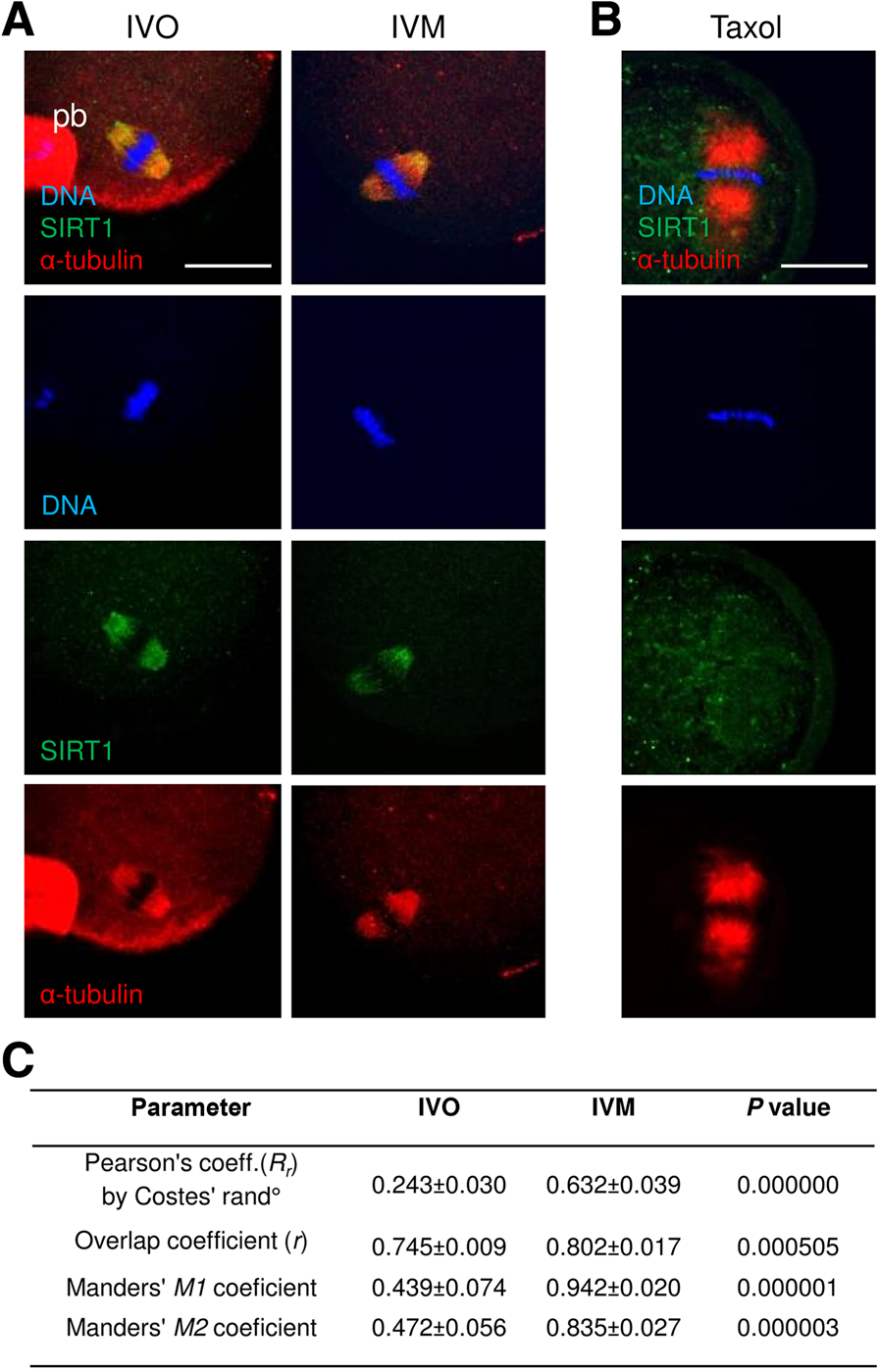
Association of SIRT1 with spindle microtubules in matured oocytes. **a** SIRT1 colocalisation with α-tubulin in IVO and IVM matured oocytes. Scale bar represents 25 μm. **b** Co-immunolabelling of SIRT1 and α-tubulin in Taxol-treated IVO oocytes. **c** The results of colocalisation analysis in IVO and IVM oocytes. JACoP (Just Another Co-localisation Plugin) approach for colocalisation of SIRT1 with the spindle α-tubulin was used [25], and sensitivity of Pearson’s *R*_*r*_ value to noise and green/red signal intensity variation was eliminated by Costes’ randomisation [26] (Costes’ rand); the Costes’ coefficient modifies Pearson’s coefficient estimating automatic threshold, eliminating false-positive colocalisation and signal noise. Manders’ overlap coefficient (*R*) was used for estimation of colocalisation. In addition, Manders’ *M1* and *M2* overlap coefficients express the proportion of green (α-tubulin), which is also red (SIRT1), and vice versa, respectively, with respect to spindle localisation as the ROI. N (number of analysed matured oocytes) = 15 per group. *t*-test was used and *P* values are indicated

Based on the SIRT1 subcellular spindle-like pattern observed in the previous experiment, we suggested the association of SIRT1 with cytoskeletal structures in mature oocytes. To support the suggestion of SIRT1-microtubule association, Taxol was used for inhibition of microtubule depolymerisation, followed by co-immunolabelling of both factors. IVO and IVM oocytes subjected to the colocalisation analysis showed a high-level overlap of SIRT1 and α-tubulin on meiotic spindles (Fig. 2a). Moreover, strong SIRT1 association with α-tubulin was detected in IVM oocytes (see Pearson’s and Manders’ coefficients, used in accordance with previous studies [25, 26]). In contrast to the SIRT1 spindle-like pattern in mature oocytes, Taxol-treated oocytes did not show SIRT1-α-tubulin association, and SIRT1 seemed to be diluted in ooplasm (Fig. 2b). The representative pictures and colocalisation coefficients are summarised in Fig. 2c.

### SIRT1 leads to the hypoacetylation of spindle α-tubulin in matured oocytes

**Fig. 3:**
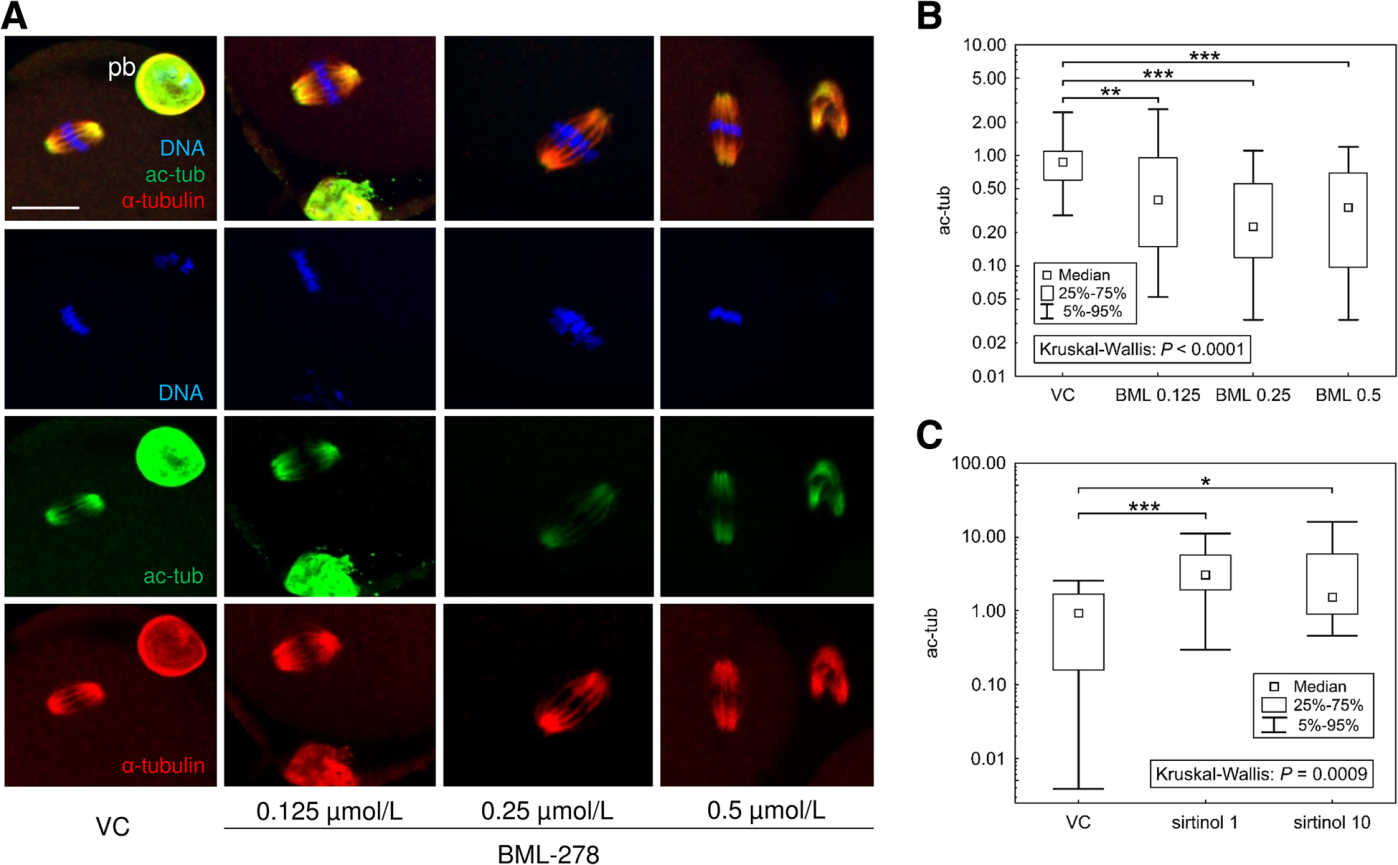
Deacetylation of spindle α-tubulin in IVM oocytes after SIRT1 activation. **a** Representative images of immunolocalised acetylated α-tubulin (ac-tub) in BML-278-treated oocytes. Scale bar represents 25 μm. **b** Image analysis of ac-tub in IVM oocytes. The quantification of integrated density parameter via ImageJ software was performed. The integrated density of BML-278-treated oocytes is normalised to the mean of the signal in vehicle control oocytes (VC) and expressed as a median ± quantiles from five independent experiments (*N* ≥ 35 per group). **c** Quantification of integrated density of oocytes treated with sirtinol (1 and 10 μmol/L), a SIRT1 inhibitor. Differences between individual group pairs were assessed post-hoc using multiple comparisons of mean ranks [30], including a Bonferroni adjustment for multiple testing; *, ** and *** denote significance at *P* ≤ 0.05, 0.01 and 0.005, respectively

Here, the selective SIRT1 activator BML-278 was used. BML-278 was assumed to be highly specific based on the provided manufacture’s informations and the biochemical studies known so far [28, 29]. First, we verified the SIRT1 activation capability of BML-278, using a SIRT1 Activity Assay Kit and comparing BML-278 with well-known non-specific SIRT1 activator resveratrol. A comparable activation ability of BML-278 was observed, and there was no significant difference in SIRT1 activity after BML-278 and resveratrol treatments (see Additional file 1: Figure S1.1). Therefore, BML-278 was used for *in vitro* treatment of mature oocytes. The oocyte maturation rate was assessed, oocytes were subsequently immunolabelled, and the acetylation of spindle α-tubulin in mature metaphase II oocytes was quantified. No effect of BML-278 on the meiosis progress and maturation rate was detected (Additional file 1: Figure S1.2). We observed the decline in acetylated α-tubulin after 0.25 μmol/L BML-278 treatment (Fig. 3b). On the other hand, sirtinol (selective SIRT1 and SIRT2 deacetylase inhibitor) increased signal intensity of acetylated α-tubulin (Fig. 3c). Based on SIRT1 and SIRT2 colocalization (Additional file 1:Figure S1.4), we consider these findings as a result of SIRT1 action. With respect to the SIRT1 pattern in mature and Taxol-treated oocytes mentioned above, we explain it by a temporary limited deacetylating action of SIRT1 on tubulin during oocyte spindle formation, rather than after spindle establishment.

### SIRT1-modulated epigenome of mature oocytes

Although SIRT1 is exclusively immunolocalised on the spindles of matured oocytes, the epigenetic SIRT1 action in oocytes is considered in accordance with our own previous findings [18]. Therefore, several post-translational histone modifications, such as positive and negative markers of genome stability, i.e., H3K9me3 [31] and H3K4me2 [32], were analysed as previously described. Moreover, we established H3K9me3 as a double-marker of SIRT1 action: i) direct histone H3 deacetylation, and ii) indirect histone H3 methylation of the same lysine residue [18]. In addition to already well-known histone markers, ubiquitinated (K119) H2A (H2AK119ub) was analysed because a SIRT1 overlap with ubiquitin-associated proteins has been reported [33]. Moreover, there have been contradictory findings of actual association of H2AK119ub with eu- or heterochromatin markers [34, 35], and H2AK119ub significance for mature oocyte quality remain unknown. Finally, the DNA protective effect of SIRT1 activator BML-278 was elucidated with a TUNEL (terminal deoxynucleotidyl transferase dUTP nick) assay.

**Fig. 4:**
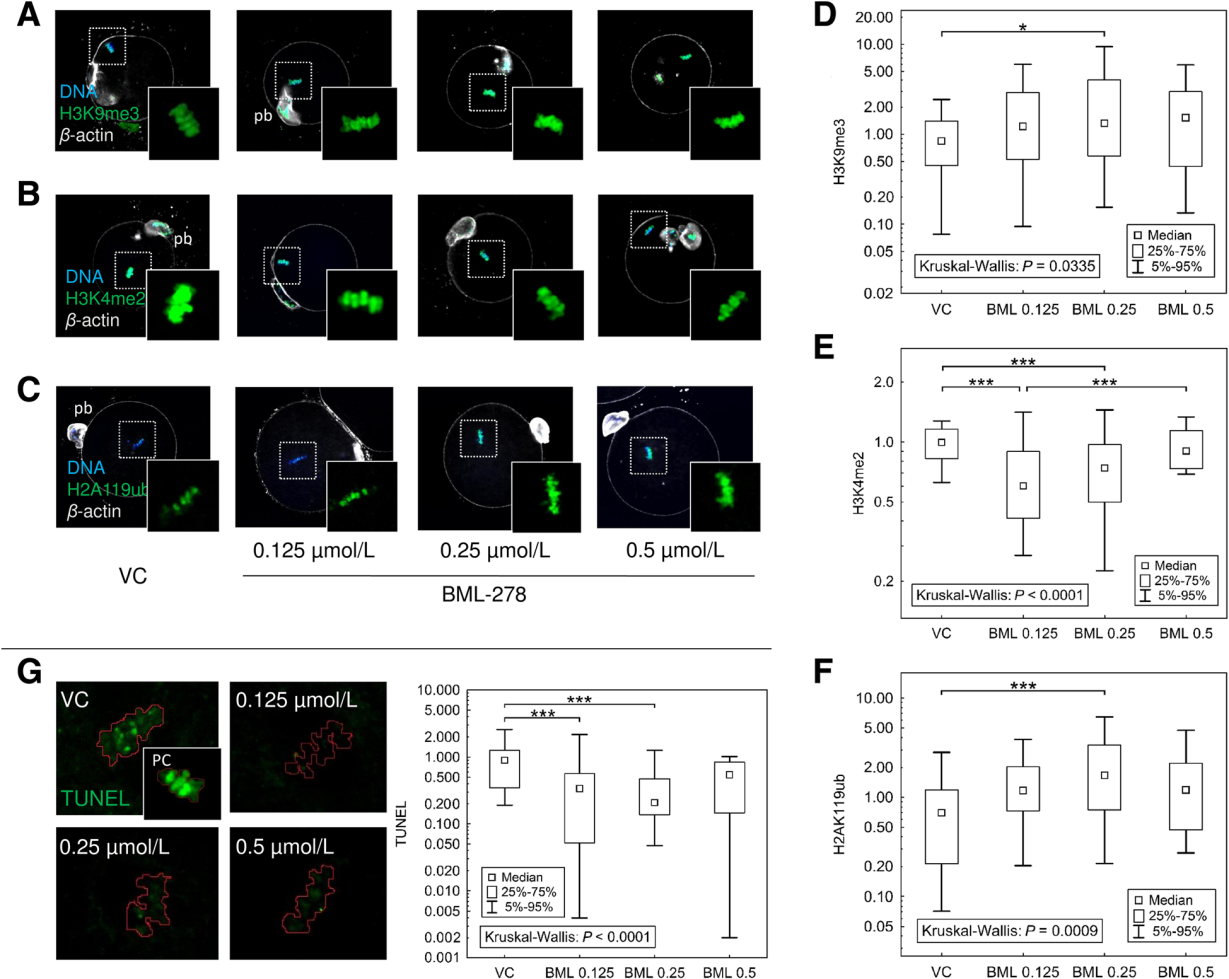
Modulated histone code in SIRT1-activated matured oocytes. **a**, **b**, **c** Representative images of H3K9me3, H3K4me2 and H2AK119ub, respectively, and the emphasis of metaphase area, used for the quantification of integrated density parameter via ImageJ software. **d**, **e**, **f** Quantification of integrated densities of H3K9me3, H3K4me2 and H2AK119ub, respectively, localised and analysed on metaphase MII chromosomes. Integrated densities are normalised to the mean of vehicle control oocytes (VC) and expressed as a median ± quantiles from five independent experiments (*N* ≥ 30 oocytes per group). **g** Effect of SIRT1 activation on DNA damage. TUNEL signal was detected in mature oocytes after BML-278 treatment, and integrated density was quantified (*N* ≥ 30 oocytes per group). The region of interest (ROI) for TUNEL signal was established in accordance with DNA staining (red line on representative pictures). TUNEL assay was elucidated using positive control (PC) through DNase I digestion of oocyte chromatin. Differences between individual group pairs were assessed post-hoc using multiple comparisons of mean ranks [30], including a Bonferroni adjustment for multiple testing; * and *** denote significance at *P* ≤ 0.05 and 0.005, respectively

The fold-change in signal intensity of trimethylation of histone H3 at lysine K9 (H3K9me3) after 0.25 μmol/L BML-278 treatment increased compared to control oocytes (Fig. 4a, d). The pericentric H3K9me3 pattern, described in previous studies [36, 37], was verified using chromosome spreading and co-staining with centromere-associated Kinesin-13 protein KIF2A (Additional file 1: Figure S1.5). In contrast, signal of dimethylation of H3 on K4 (H3K4me2) was significantly decreased to 0.69 and 0.74 after 0.125 and 0.25 μmol/L BML-278 treatments, respectively (Fig. 4b, e). The increase in H2AK119ub was etected after 0.25 μmol/L BML-278 treatment (2.46 ± 0.33 versus 1.0 ± 0.17; Fig. 4c, f), consistent with the H3K9me3 heterochromatin marker. The DNA protective effect of BML-278-activated SIRT1 was assessed through TUNEL assay. In accordance with H3K9me3 and H3K4me2, we observed decreasing integrated TUNEL density in oocytes matured in the presence of BML-278 (0.125 and 0.25 μmol/L), compared to control oocytes (0.36–0.47 vs. 1.0; Fig. 4g).

Our results showed significant changes in signal intensities of the fluoresceins staining individual histone PTMs. These findings point out the SIRT1-shifted histone code and chromatin quality of matured oocytes after BML-278 treatment. Moreover, the heterochromatin-associated ubiquitination of H2A, rather than as a DNA damage marker is strongly indicated. Although SIRT1 lost the association with chromatin as soon as NEBD occurred, we rendered SIRT1-driven chromatin quality in matured MII oocytes, and therefore, we assumed that SIRT1 modulates histone code in immature GV oocyte because SIRT1 is exclusively localised in GVs.

### SIRT1 drives histone code establishment in immature GV oocytes

**Fig. 5:**
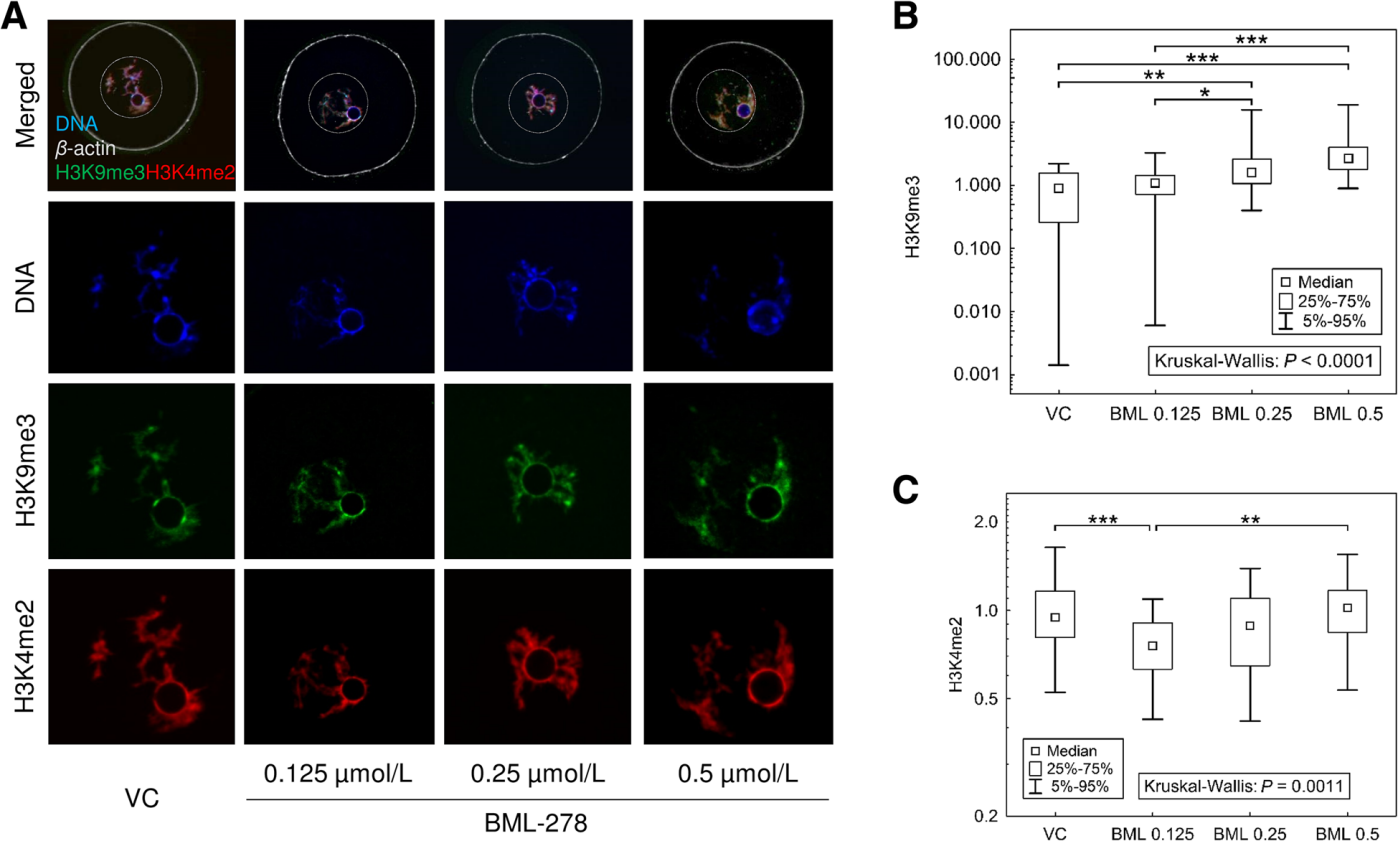
Modulated histone code in BML-278-treated immature GV oocytes. **a** Representative images of H3K9me3 and H3K4me2 subjected to the quantification of integrated density parameter via ImageJ software. **b**, **c** Quantification of integrated density of H3K9me3 and H3K4me2 localised and analysed in germinal vesicle (GV) of oocytes treated with BML-278 activator, respectively. The integrated density of BML-278-treated oocytes is normalised to the mean of the signal in control oocytes and expressed as a median ± quantiles from five independent experiments (*N *≥ 30 oocytes per group Differences between individual group pairs were assessed post-hoc using multiple comparisons of mean ranks [30], including a Bonferroni adjustment for multiple testing; *, ** and *** denote significance at *P* ≤ 0.05, 0.01 and 0.005, respectively

In this experiment, GV oocytes were kept under meiosis-suppressing conditions for 16 h and treated with SIRT1 activator BML-278. With respect to the exclusive SIRT1 location in GVs, we assumed histone targets of SIRT1 in GV immature oocytes. To test this suggestion, we used previously introduced histone markers, and the integrated densities of H3K9me3 and H3K4me2 were analysed. We observed an increase in the signal intensity of H3K9me3 after 0.25 μmol/L and 0.5 μmol/L BML-278 (2.91 ± 0.83 and 4.84 ± 1.16, respectively, vs. 1.0 ± 0.15 in control). In contrast, these BML-278 doses had no effect on H3K4me2, however, 0.125 μmol/L BML-278 treatment decreased the signal intensity of H3K4me2 (Fig. 5). Based on the observed effect of BML-278 treatment on signal intensities of both histone PTMs, we can consider SIRT1 epigenetic mode of action in immature GV oocytes and a different, rather non-epigenetic, molecular mechanism in mature oocytes. These findings are in accordance with a previous observation, where SIRT1 was lacking in the perichromatin area immediately after NEBD, and colonised spindles in matured MII oocytes.

## Discussion

Sirtuins represent a potent group of proteins relevant to several fields of medical studies, including both veterinary reproduction and human assisted reproduction technologies [23, 38–40]. Indeed, in accordance with previous studies [41–43], we detected SIRT1 in both immature GVs and matured metaphase II (MII) oocytes through different approaches. In addition to SIRT1 detection, the possibility of modulation of SIRT1 activity due to pharmacological treatment offers many medical implications. Therefore, we have chosen BML-278, a SIRT1 specific activator with only minor ability to activate SIRT2 and SIRT3. Based on recent knowledge, BML-278 is the most selective activator for SIRT1 [28, 29]. Moreover, observed phenotypes are considered to be a result of SIRT1 action with respect to different subcellular localisation of SIRT1 and SIRT2.

Our observations reveal SIRT1 to be present exclusively in GV of immature oocytes; however, the SIRT1 signal is diluted in the ooplasm immediately after nuclear envelope breakdown (NEBD), and finally forms a spindle-like pattern in matured MII oocytes, comparable with other histone deacetylases in oocytes [44–46] and SIRT1 in human somatic cells [47]. Based on this finding, we have elucidated the deacetylating action of SIRT1 towards spindle tubulin in matured MII oocytes that was proved by sirtinol (a SIRT1 deacetylase inhibitor) treatment. Our study, utilising colocalisation analysis, quantified SIRT1 association with the spindle α-tubulin and revealed the overlap of both factors in *in vivo* and *in vitro* matured oocytes. This observation is in accordance with the previously described involvement of HDAC3 and HDAC8 in deacetylation of spindle tubulin, which, therefore, is responsible for microtubule attachment to the kinetochore and euploidy maintenance in matured oocytes [44, 45]. Even, SIRT2 has been found on the spindle, however, in contrast to our observation, SIRT2 occupies uniquely metaphase I spindle [48]. Surprisingly, the deacetylating action of HDACs on the spindle tubulin is considered a phenomenon essential for metaphase II spindle assembly in oocytes [44, 46], although tubulin acetylation is a marker of stable microtubules [49]. On the other hand, SIRT1 may contribute to microtubule polymerisation via alternative PLK1 regulation [50] through equal localisation on the oocyte spindle.

The SIRT1 spindle pattern observed in mature oocytes seems to be a result of successive SIRT1 re-localisation and short-term spindle occupation. This suggestion is supported by i) almost no observable signal in metaphase I oocytes and subsequently, a gradual spindle-like pattern of SIRT1 during meiosis progression, ii) no association of SIRT1 with overpolymerised α-tubulin in Taxol-treated oocytes, and iii) weaker association of SIRT1 in *in vivo *mature oocytes. Accordingly, *in vivo* mature oocytes represent a physiological control for *in vitro* experiments, and lower colocalisation coefficients underline decreasing SIRT1 requirements on oocyte spindles after metaphase II achievement. Based on the findings in *in vivo* matured oocytes and Taxol-treated oocytes, declining SIRT1-α-tubulin association suggests a preparation of the spindles of mature oocytes for subsequent changes following fertilisation [51]. Moreover, other SIRT1-associated proteins and potent deacetylating targets (e.g., transcriptional factors, core histones) are worth considering [18, 52, 53], and a complex physiological role of the SIRT1 action through these substrates remains to be elucidated.

Because of the well-known SIRT1 targets leading to modulation of the epigenetic code [18, 33], we studied several histone modifications in mature MII oocytes treated after SIRT1 activation. In accordance with the aforementioned postulation of many SIRT1 targets in the oocyte, we revealed the SIRT1-shifted histone code towards chromatin stabilisation and DNA protection, in accord with other sirtuins [54, 55]. We may consider both epigenetic and non-epigenetic substrates in oocyte epigenome modulation, such as lysine K9 in histone H3 deacetylated through SIRT1 and enzymes catalysing methylation of core histones, respectively. Considering histone methylation, increasing activity of SUV39H1 histone methyltransferase is suggested, in accordance with previous observation [22], to result in increased H3K9me3 [56]. In contrast, histone demethylase LSD1/KDM1A demethylating H3K4 is a plausible substrate of SIRT1 in mouse oocytes [21]. Therefore, SIRT1 can achieve significant impact through the regulation of LSD1/KDM1A in gametes as well as early embryos and embryonic stem cells [57, 58].

In addition to H3K9me3 and H3K4me2, we elucidated the ubiquitination of H2A at lysine K119 (H2AK119ub). In our experiment, BML-278 treatment increases H2AK119ub signal although decreasing occurrence is assumed, in accordance with the knowledge of ubiquityl-H2A accompanying DNA damage [35, 59]. However, there is also an evidence of H2AK119ub to be heterochromatin repressive mark [34, 60]. In accordance with these studies, we consider an involvement of SIRT1 in ubiquitin-proteasomal system-modulated chromatin, which is consistent with our earlier findings of MDM2 ubiquitin E3 ubiquitin ligase interaction with SIRT1 [18]. A dual physiological role of H2AK119ub seems to be heterochromatin-marking and DNA protection in mature oocytes, however, a comprehensive study is required for testing of this hypothesis.

The above-mentioned histone PTMs occur in mature oocytes, although SIRT1 subcellular localisation is not associated with condensed chromosomes. These facts lead us to postulate an inheritance of histone modifications acquired earlier than oocytes mature. Therefore, we tested post-translational changes of histone H3 in GV oocytes after SIRT1 activation via BML-278. Indeed, GV histone H3 is modified at lysine K9 in a SIRT1-dependent manner, favouring heterochromatin features for gene silencing, chromatin stability and DNA protection [61, 62]. In addition to H3K9me3, H3K4me2 shows a decrease after 0.125 μmol/L BML-278 treatment of GV oocytes. Hence, we suggest that the SIRT1-modulated histone code, observed in mature oocytes, is attained earlier and inherited from the GV stage. Furthermore, the involvement of LSD1/KDM1A demethylase may be considered in SIRT1-driven modulation of H3K4me2 in GV and MII oocytes [21]. This assumption is supported by the observation of LSD1 spindle-like distribution in somatic cells [63], and we can surmise the SIRT1-LSD1 crosstalk resulting in modulation of H3K4me2 in mature oocytes.

SIRT1 seems to be capable of both epigenetic and non-epigenetic mode of molecular action, in immature GV oocytes and matured MII oocytes, respectively. Accordingly, immature GV oocytes, arrested in the first meiotic arrest, tender more available chromatin for epigenetic modulators in extensive time window, including oocyte growth [64, 65]. On the other hand, mature oocytes arrested at a time-limited stage of metaphase II and containing highly condensed chromatin offer fewer opportunities for epigenetic modifications. The ability to switch the epigenetic and non-epigenetic mode of action during oocyte maturation is proposed and the mechanism of this exchange is a subject of further study.

In accordance with the epigenetic to non-epigenetic switch assumption, anti-SIRT1-immunodetected 60-kDa protein (supposed SIRT1 isoform 2) shows a shift in molecular weight of this protein towards 65-kDa with oocyte maturation. The change indicates an achievement of post-translational modification [66–68] and/or protein-protein interaction [69], in mature MII oocytes. Based on our best knowledge, we presumed a crosstalk of SIRT1 and the ubiquitin-proteasomal system [33, 66]. A description of SIRT1-interacting proteins and clarification of the physiological role of SIRT1 PTMs in oocyte maturation, fertilisation and early embryonic development remains to be elucidated.

## Conclusions

Our results show that SIRT1 is predestined for an epigenetic mode of action in immature GV oocytes while SIRT1 distributes in a spindle-like pattern in fully mature oocytes where SIRT1-decreased tubulin acetylation occurs. Our observations suggest a dual spatiotemporal SIRT1 action in oocytes and the capability of being readily switched during the meiosis progress is indicated.

## Additional file

Additional file 1: **Figure S1.1.** The effect of BML-278 on SIRT1 activity. **Figure S1.2.** The results of oocyte maturation after BML-278 treatment. **Figure S1.3.** The full-length blot of SIRT1 and β-actin (loading control). **Figure S1.4.** SIRT2 expression in oocytes and colocalisation with SIRT1. **Figure S1.5.** H3K9me3 and KIF2A co-immunostaining of chromosome spread. (DOCX 2033 kb)

## Data Availability

Please contact author for data requests.

## Abbreviations

BML-278: a selective SIRT1 activator
GV: Germinal vesicle
H2AK119ub: Ubiquitinated histone H2A on lysine K119
H3K16ac: Acetylated histone H3 on lysine K16
H3K4me2: Di-methylated histone H3 on lysine K4
H3K9me3: Tri-methylated histone H3 on lysine K9
HDAC: Histone deacetylase
IBMX: 3-Isobutyl-1-methylxanthine
IVM: *In vitro* maturation (of oocytes)
IVO: *In vivo* ovulated (oocytes)
KIF2A: Kinesin family member 2A, centromere-associated kinesin-like protein
LSD1/KDM1A: Lysine-specific histone demethylase 1A/lysine (K)-specific demethylase 1A
MDM2: Mouse double minute 2 homolog, E3 ubiquitin-protein ligase
MII: Metaphase II (oocytes)
NEBD: Nuclear envelope breakdown
PLK1: Polo-like kinase 1, serine/threonine-protein kinase 1
SIRT, Sirtuin: Silent mating type information regulator 2 homolog, NAD^+^-dependent histone deacetylases
SUV39H1: Suppressor of variegation 3-9 homolog 1, histone-lysine N-methyltransferase
TUNEL: Terminal deoxynucleotidyl transferase (TdT) dUTP nick-end labeling assay
