# Supplementary material for: Epigenetic and non-epigenetic mode of SIRT1 action during oocyte meiosis progression
Source: J Anim Sci Biotechnol. 2019 Aug 9;10:67. doi: 10.1186/s40104-019-0372-3 (PMC6688279; doi:10.1186/s40104-019-0372-3)
Supplement: Supplementary file 1 — Figure S1.1. The effect of BML-278 on SIRT1 activity. Figure S1.2. The results of oocyte maturation after BML-278 treatment. Figure S1.3. The full-length blot of SIRT1 and β-actin (loading control). Figure S1.4. SIRT2 expression in oocytes and colocalisation with SIRT1. Figure S1.5. H3K9me3 and KIF2A co-immunostaining of chromosome spread. (DOCX 2033 kb) [file 40104_2019_372_MOESM1_ESM.docx]

Additional file 1

Epigenetic and non-epigenetic mode of SIRT1 action during oocyte meiosis progression

Jan Nevoral^1,2^*, Lukas Landsmann^1,3^, Miriam Stiavnicka^1^, Petr Hosek^1^, Jiri Moravec^1^, Sarka Prokesova^1,4^, Hedvika Rimnacova^1^, Eliska Koutna^1,4^, Pavel Klein^1^, Kristyna Hoskova^5^, Tereza Zalmanova^5^, Tereza Fenclova^1^, Jaroslav Petr^5^, Milena Kralickova^1,2^

^1^Biomedical Center, Faculty of Medicine in Pilsen, Charles University, Pilsen, Czech Republic

^2^Department of Histology and Embryology, Faculty of Medicine in Pilsen, Charles University, Pilsen, Czech Republic

^3^Faculty of Science, Charles University, Prague, Czech Republic

^4^Faculty of Agriculture, Food and Natural Resources, Czech University of Life Sciences in Prague, Czech Republic

^5^Institute of Animal Science, Prague 10-Uhrineves, Czech Republic

*Corresponding author: [jan.nevoral@lfp.cuni.cz](mailto:jan.nevoral@lfp.cuni.cz)

Content

**Figure S1.1** The effect of BML-278 on SIRT1 activity.

**Figure S1.2** The results of oocyte maturation after BML-278 treatment.

**Figure S1.3** The full-length blot of SIRT1 and β-actin (loading control).

**Figure S1.4** SIRT2 expression in oocytes and colocalisation with SIRT1.

**Figure S1.5** H3K9me3 and KIF2A co-immunostaining of chromosome spread.

**Figure S1.1** The effect of BML-278 on SIRT1 activity. Pure SIRT1 protein was treated in vitro and the assay was performed in accordance with manufacture protocol (fluorometric SIRT1 Activity Assay Kit; Abcam, Cambridge, UK, ab156065). The activity of BML-278 was related to resveratrol (RES), a non-selective activator of sirtuins, used as a positive control.

**Figure S1.2** The results of oocyte maturation after BML-278 treatment. Based on the verification of the capability of BML-278 to activate SIRT1, oocytes were treated with BML-278 during in vitro maturation. No significant differences were observed. GV: germinal vesicle stage (immature oocytes), MI: metaphase I oocyte, MII: metaphase II oocyte (matured oocyte with extruded polar body). Dg: degenerated oocytes (i. e. apoptosis, lysis).


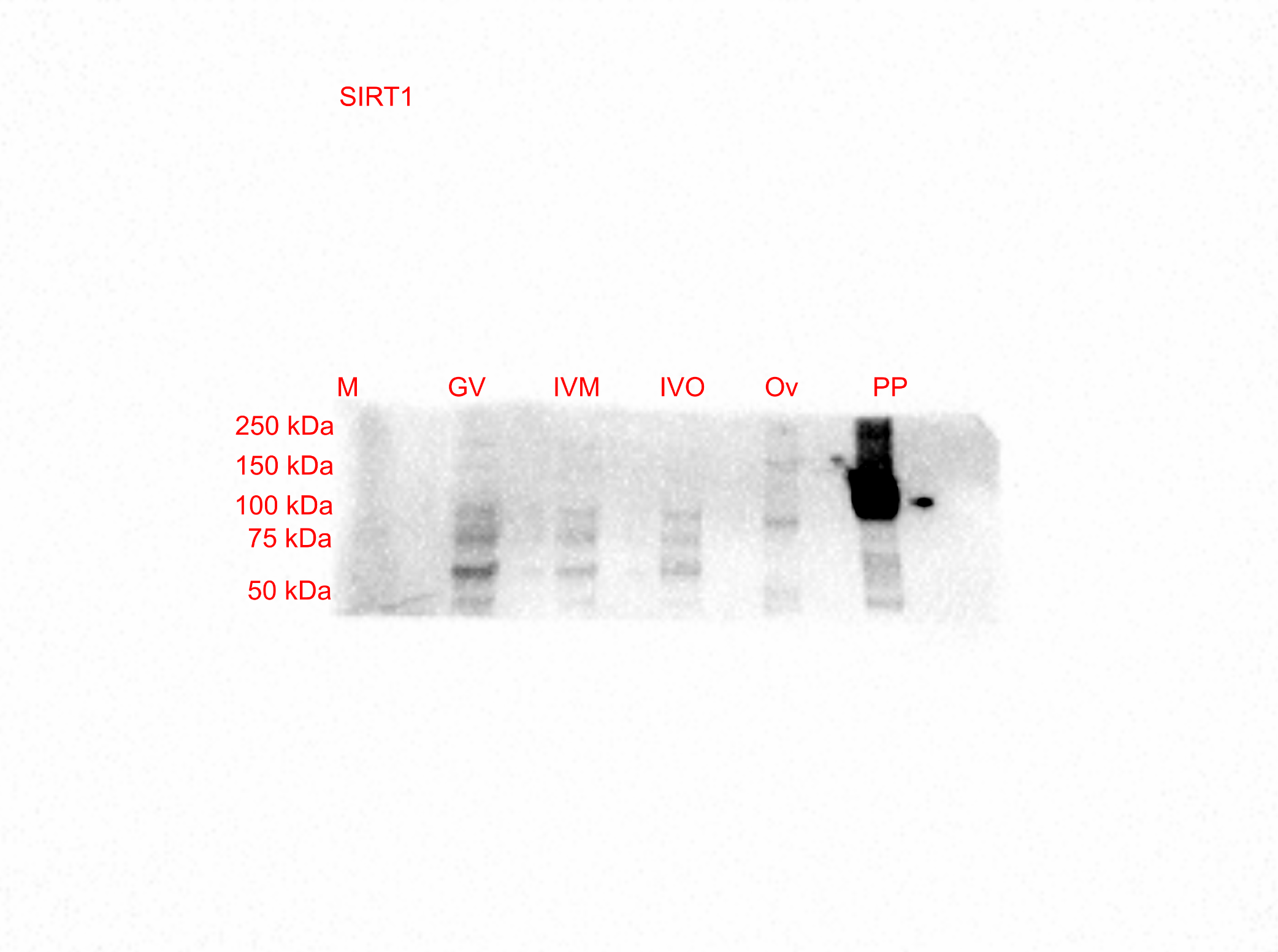


**
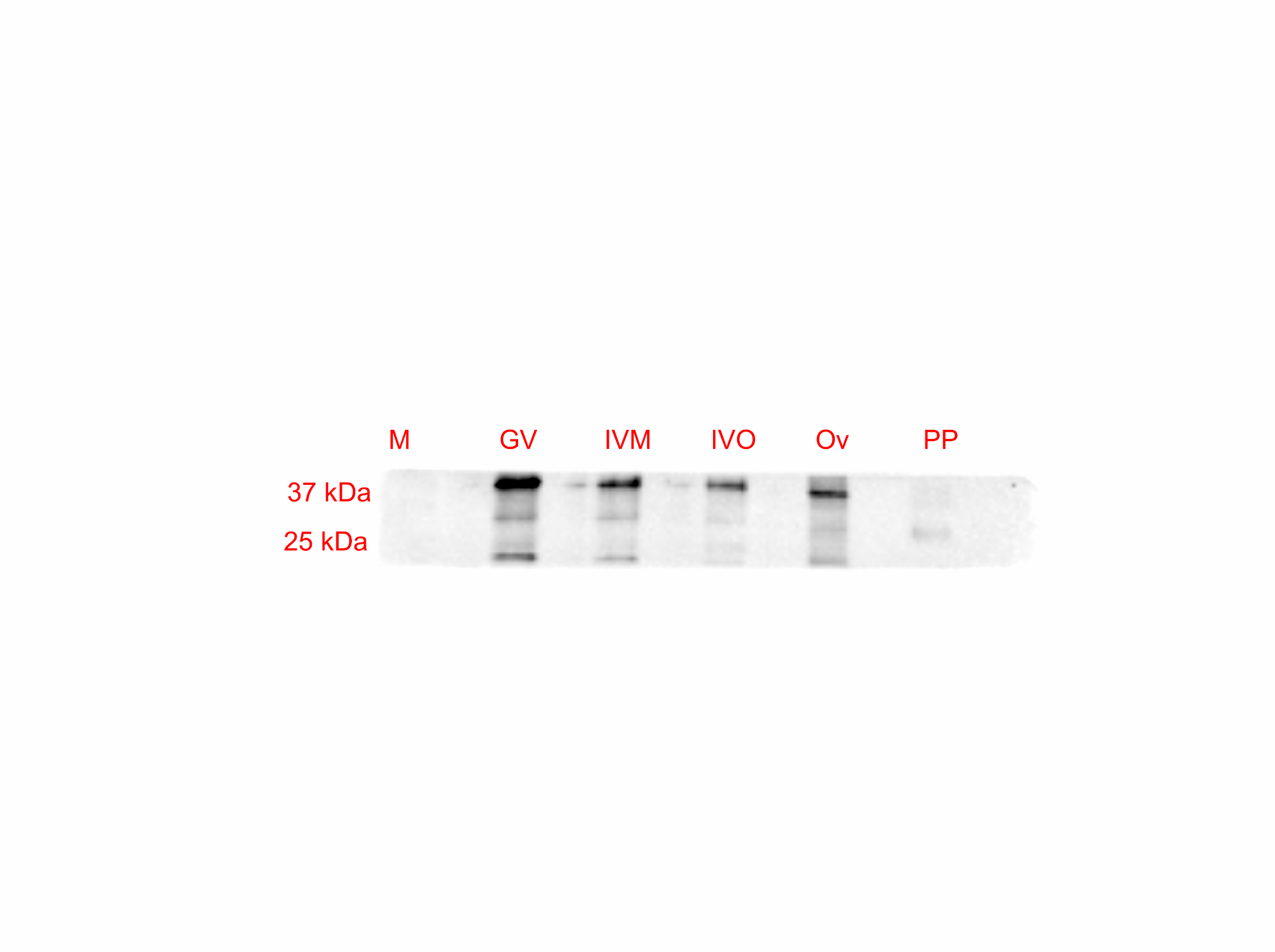
**

**Figure S1.3** The full-length blot of (A) SIRT1 and (B) β-actin (loading control). M: molecular weight marker, GV: germinal vesicle oocytes, IVO: in vivo matured oocytes, IVM: in vitro matured oocytes (approx. 200 oocytes per lane), Ov: ovarian tissue lysate (approx. 10 µg of total proteins in the lane), PP: pure SIRT1 protein.


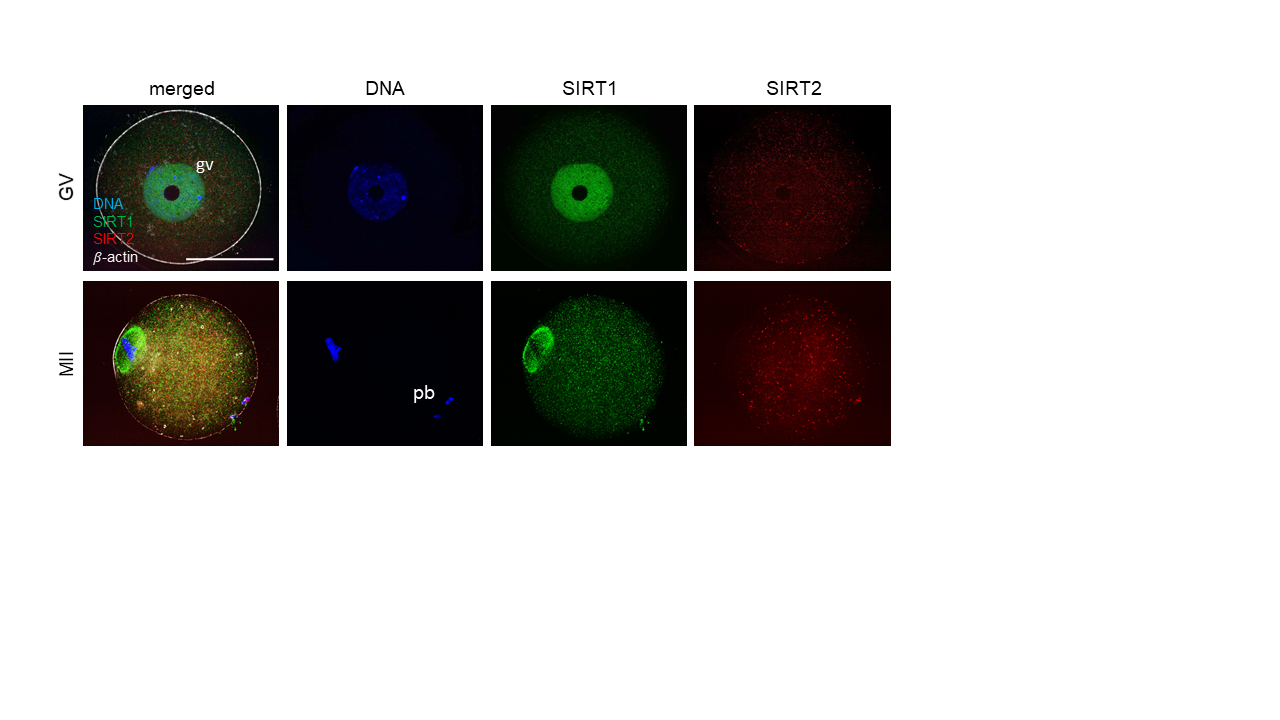


**Figure S1.4** SIRT2 expression in immature GV and *in vitro* matured MII oocytes, and colocalisation with SIRT1. gv: germinal vesicle, pb: polar body. Scale bar represents 50 µm.


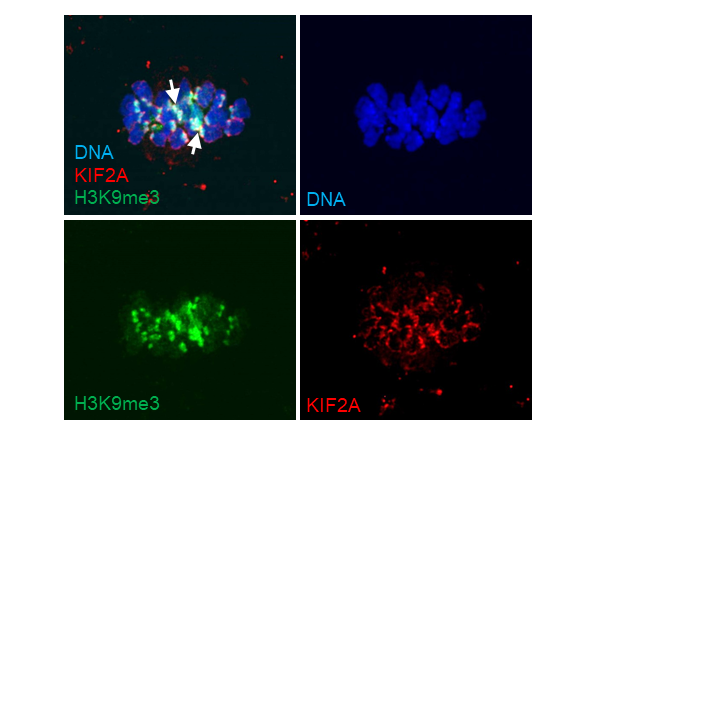


**Figure S1.5** H3K9me3 and KIF2A co-immunostaining of chromosome spread. Arrow indicates the overlay of the methylation of histone H3 on lysine K9 (H3K9me3) and Kinesin family member 2a (KIF2A), a centromere-associated protein.
